# The metabolic efficiency of myelinated vs unmyelinated axons

**DOI:** 10.1186/1471-2202-12-S1-P100

**Published:** 2011-07-18

**Authors:** Ali M Neishabouri, Aldo A Faisal

**Affiliations:** 1 Department of Bioengineering, Imperial College London, London, SW7 2AZ, UK; 2 Department of Computing, Imperial College London, London, SW7 2AZ, UK

## 

Understanding the metabolic efficiency of axons is of critical importance in neuroscience: such as interpreting fMRI and other imaging data, understanding the impact of metabolic conditions and anoxia/hypoxia on the CNS, and finally because current data suggests that metabolic cost places severe limits on neuronal activity[1]. Surprisingly, the metabolic cost of action potential (AP) in myelinated nerve fibers has not been directly estimated, unlike the cost of unmyelinated axons[1,2]. We calculated the metabolic cost of saltatory APs using detailed stochastic, biophysical models of a human myelinated peripheral axon and compared it to APs in unmyelinated axons of equal diameter. We modelled both axon types using the Modigliani stochastic simulator[3,4]. For the human myelinated axon, we modelled a heterogeneous multi-compartment cable of 3 cm length. Each Node of Ranvier is surrounded by paranodal regions and separated by a myelinated internode. Ion channel kinetics and axonal geometry were taken from published physiological and anatomical data[5]. Parameters for the unmyelinated axon were taken from[6]. We measured the metabolic cost of APs as the amount of ATP needed for Na-K-pumps to restore ion concentrations after an AP [1,2,8].

This allowed us to systematically investigate how metabolic cost depends on factors such as axonal geometry and ion channel densities. E.g comparing myelinated and unmyelinated axons with the same axon diameter of 1µm (fibre diameter including myelin sheath was 3.7 µm) we find the following: single APs at a myelinated axon’s Node of Ranvier have a metabolic cost of 3.5 pmol/cmÂ² ATP per unit membrane area. This is approximately 7 times the amount per AP in hippocampal mossy fibre (0.53 pmol/cm^2^;[2]) but less than leaky squid axons (5 pmol/cm^2^). However, Node of Ranvier cover only 0.33% in our myelinated axon. The internodal regions contain hardly any Na^+^ channels, but a low density of K^+^ channels (3 µm^-2^) along internodes, and higher densities at the paranode (80 µm^-2^). We estimate overall energy consumption based on Na currents at the Node and K along a segment comprising half the internodal fibre on both sides of a node. This yields an AP cost per myelinated axon segment of 0.05 pmol/cm^2^ ATP per unit membrane area. Thus, per unit length the myelinated axon is 70 times metabolically cheaper than mossy fibers. The added bulk of the myelin allows 13 unmyelinated axons of same diameter to fit in the volume of one myelinated fibre, suggesting that the ratio of energy demand over wiring density is 5 times higher for myelinated fibers vs unmyelinated axons.

Our findings are novel, as the costs of saltatory APs were not calculated from the bottom-up before. They are important, as it was generally assumed that myelinated axons evolved mainly for speed of propagation[7] at the cost of achievable wiring density[6,8], but energy may be as important: in cortical grey matter unmyelinated axons operate close to the noise limits to wiring miniaturisation[6], yet still contains myelinated axons[9]. We can explain this with a four-way trade-off between time delay, metabolic cost, volume constraints and noise limits to axonal anatomy.
